# Hormesis and Low Toxic Effects of Three Lanthanides in Microfungi Isolated from Rare Earth Mining Waste in Northwestern Russia

**DOI:** 10.3390/toxics11121010

**Published:** 2023-12-10

**Authors:** Elena A. Kasatkina, Oleg I. Shumilov, Irina Y. Kirtsideli, Dmitry V. Makarov

**Affiliations:** 1 Institute of North Industrial Ecology Problems, Kola Science Centre, Russian Academy of Sciences, 184209 Apatity, Russia; o.shumilov@ksc.ru (O.I.S.); d.makarov@ksc.ru (D.V.M.); 2 Komarov Botanical Institute, Russian Academy of Sciences, 197376 Saint Petersburg, Russia; microfungi@mail.ru

**Keywords:** rare earth elements, low-dose toxicity, microfungi, hormesis, mine tailings, Arctic

## Abstract

The low-dose toxicity of chloride and nitrate salts of three lanthanides (La, Ce and Nd) was tested on six microfungal species. Five of them (*Geomyces vinaceus*, *Aspergillus niveoglaucus*, *Pseudogymnoascus pannorum*, *Penicillium simplicissimum* and *Umbelopsis isabellina*) were isolated from the loparite ore tailings on the Kola Peninsula, northwestern Russia. *Sydowia polyspora* was a control strain. In the case of nitrate salts, the toxicity of REEs to four of six microorganisms was significantly (*p* < 0.5) lower compared to chloride salts. In this case, nitrates can play the role of exogenous nutrients, compensating for the toxic effect of REEs. Interestingly, *U. isabellina* only showed an opposite response, indicating the highest toxicity of nitrate (IC_5_ = 9–20 mg/L) REEs’ salts compared to chlorides (IC_5_ = 80–195 mg/L) at low concentration levels. In addition, treatment with lanthanides showed a “hormesis effect” on fungal growth with stimulation at low doses and inhibition at high doses. However, *U. isabellina* and *S. polyspora* demonstrated the absence of hormetic response under the treatment of REEs’ nitrate salt. Taking into account the specific hormetic responses and high tolerance of *P. simplicissimum* and *U. isabellina* to lanthanides, our findings may be useful in the assessment of the potential application of the selected fungi to bioremediation and REE bioleaching.

## 1. Introduction

The use of rare earth elements (REEs) in many sectors of the world economy (e.g., agriculture, medicine, industry) is constantly growing. The REEs include the elements scandium (Sc), yttrium (Y) and fourteen lanthanides following lanthanum (La): cerium (Ce), praseodymium (Pr), neodymium (Nd), promethium (Pm), samarium (Sm), europium (Eu), gadolinium (Gd), terbium (Tb), dysprosium (Dy), holmium (Ho), erbium (Er), thulium (Tm), ytterbium (Yb) and lutetium (Lu). Since REEs are extremely important in high-tech and green energy industries as a raw material for the production of superconductors, autocatalytic converters, mobile phones, supermagnets, LED lightings, smart-batteries, solar panels and wind turbines, these elements are called the “vitamins of industry” [1]. Moreover, REEs are used in agriculture as fertilizers for improving crop growth and production [1,2,3,4]. Recently, the European Union banned all in-feed growth-promoting antibiotics; thereafter, some REEs have been introduced as alternative feed additives promoting the growth and productivity of farm animals [5]. Additionally, several studies confirmed REE-associated antioxidant and antiseptic effects in the treatment of some diseases [6].

The growing use of REEs in the economy has caused an explosive increase in rare earth mining activities [1,7]. Therefore, there is an imminent anthropogenic emission of these elements into the surrounding media from different sources, e.g., mining enterprises, mining and industry wastes and fertilizers [1]. As a result, the anthropogenic anomalies of REEs in the soil, water ecosystems and sediments have already been registered [2,8,9,10,11,12,13]. Thus, REEs may now be considered as possible pollutants with potential toxic effects on the environment [1,3]. However, information on ecotoxicity, environmental concentrations, hazardous levels and bioaccumulation properties of REEs is still insufficient [1,14,15]. During the last decades, it has been reported that increasing rare earth concentrations in the environment may cause their accumulation in different groups of organisms, including animals and humans [5,6,7,14,15]. People working in the rare earth industry and living in mining areas could be exposed to REEs via different pathways, e.g., inhalation from air or ingestion from food and drinking water [6,14,15,16,17,18,19]. Specifically, an increased REE content in the hair of workers engaged in solar cell production has recently been reported [19]. A few reports regarding occupational REE exposure have indicated adverse health effects on the respiratory tract, that is, the development of pneumoconiosis through rare earth bioaccumulation [14,18]. For instance, La interacts significantly with erythrocytes and leukocytes, inhibiting its normal function and recovery [20]. Some studies have reported that REE at a low dose may have beneficial effects that are termed hormesis, and at a high dose, they may have harmful effects on the growth and development of plants [21,22,23], bacteria [24], microfungi [25,26,27,28], aquatic organisms [29] and animal models [30,31]. Despite the fact that hormesis has been a well-known phenomenon for a long time [32,33,34], there are few studies concerning the response of microorganisms, including fungi, to rare earths at hormetic doses [24,25,26,27,28]. Therefore, questions concerning toxic and favorable REE-associated effects are still important research subjects among scientists [1,6,14,15,35]. Since information regarding rare earth toxicity in biota and humans is still scarce, further toxicological studies of these elements are needed, e.g., based on bioassay methods [35,36,37,38].

The traditional methods of REE mining are highly expensive and involve the use of toxic chemicals (nitric and sulfuric acids), resulting in negative environmental impact [1,7]. These reasons, in addition to the limited global reserves of REEs, led the industry to search for alternative REE resources, including tailings, electronic and fertilizer wastes [2,7,11,39,40] and eco-friendly, nature-like methods of rare metal extraction, e.g., bioremediation and bioleaching [7,38,40,41]. Russia, which accounts for 13.6% of the world’s REE reserves [1], currently exploits the only active deposit of loparite ores, the Lovozero deposit, located behind the Arctic Circle on the Kola Peninsula, northwestern Russia [11]. Additionally, several large mining and procession enterprises concentrated on the Kola peninsula (e.g., JSC Apatit, Lovozersky GOK, Kovdorsky GOK, Olenegorsky GOK, North-Western Phosphorous Company) are powerful sources of negative environmental impact [10,11,12,13,42]. Another main resource of rare metals is apatite–nepheline ores from the Khibiny deposits, Kola Peninsula [2,11]. These deposits were developed by JSC Apatit and North-Western Phosphorous Company [42]. The main products are apatite concentrate and phosphate fertilizers containing up to ~2600 mg/kg of REEs [2]. Due to these very high concentrations of REEs in the Kola superphosphates, these phosphate fertilizers can be considered promising alternative REE resources [2,7,40,41]. The recovery of REEs from loparite and apatite–nepheline tailings helps sustainable development through the circular economy and by creating new nature-like technologies. The mining and processing of regional minerals is the main source of the REE anthropogenic anomalies registered in the soils, water bodies and sediments on the Kola Peninsula [10,11,12,13]. The main potential risks of regional environmental toxicity are the mine tailings [11,12,13]. Thus, the average content of REEs in the surface layers of Lake Lovozero reaches 736 mg/kg [13]. The average concentrations of REEs in the Kola loparite and apatite–nepheline ore tailings are 1150 mg/kg and 770 mg/kg, respectively; therefore, these mining wastes can become sources for the extraction of REEs [11]. Recent studies investigated the biological potential of some microfungi to recover REEs from the Kola loparite ore tailings [26,38].

To perform an adequate evaluation of the potential hazards of REEs, more data on the toxicity of individual rare metals in different test conditions is needed. The main aims of this study are (a) to assess the potential low toxic and hormetic effects of three lanthanides (La, Ce, Nd), as well as to some microfungi isolated from the loparite ore tailings, and (b) to compare the toxicity of individual elements in the form of chloride and nitrate salts.

## 2. Materials and Methods

### 2.1. Microfungal Strains and Chemical Reagents

The microfungal strains used in the present study were *Aspergillus niveoglaucus* OQ165229; *Geomyces vinaceus* OQ165231; *Penicillium simplicissimum* OQ165232; *Pseudogymnoascus pannorum*; *Umbelopsis isabellina* OQ165236 isolated from the Loparite tailing dump of the Lovozersky Mining and Processing Plant (MPP) (67.9° N, 34.6° E) [26,38]. Another strain was *Sydowia polyspora* supplied by the Komarov Botanical Institute, Saint Petersburg, Russia. The last two fungi are typical subarctic representatives [26,43].

The lanthanides La^3+^, Ce^3+^ and Nd^3+^ were tested in the form of their nitrates and chlorides: lanthanum nitrate hexahydrate La(NO_3_)_3_·6H_2_O (>99%), cerium nitrate hexahydrate Ce(NO_3_)_3_·6H_2_O (>99%), neodymium nitrate hexahydrate Nd(NO_3_)_3_·6H_2_O (>99%), cerium chloride heptahydrate CeCl_3_·7H_2_O (>99.5%) and neodymium chloride hexahydrate NdCl_3_·6H_2_O (>99.5%).

A rare earth nitrate form was used in this study since this is the common elemental form present in REE-enriched fertilizers [27].

### 2.2. REE Toxicity Test

Toxicity levels of lanthanides (La^3+^, Ce^3+^, Nd^3+^) were assessed. REE stock solutions with different concentrations (0–10,000 mg/L) were prepared by dissolving their nitrate and chloride salts with sterilized water. The media were adjusted to the same pH value (6.5–6.8). Strains were then inoculated on Sabouraud dextrose agar (SDA) with three replicates. The control strain was inoculated on SDA without REEs. The strains were cultured on Petri plates (diameter 90 mm) in the dark at 20 °C until the colony reached the edge of the plate. Colony diameters were measured daily. At the end of experiment, changes in macro- and micromorphology were registered. The inhibition ratios IR (%) of lanthanides against the microfungi were calculated:

IR (%) = (*D*_c_ − *D*_t_)/(*D*_c_ − *D*_0_)·100%, (1)

where *D*_c_ is the diameter of control colony without REEs (cm), *D*_t_ is the diameter of REE REE-exposed colony (cm) and *D*_0_ is the initial colony diameter (cm).

For cluster analysis, the results of REE-growth inhibition for all six fungi were combined and compared. The data were examined using the nearest-neighbor clustering method. Euclidean distance was used to combine clusters and construct a dendrogram.

To estimate low toxic (IC_5_), median (IC_50_) and hormetic effects of lanthanides, we used the regression-based approach [44,45]. IC_5_ and IC_50_ are the estimated concentrations resulting in 5% and 50% inhibition of fungal growth relative to the control after a specified exposure time, respectively [44]. Below 5% effect, the confidence intervals become excessively large, and estimates of low toxic effects are largely model-dependent [45]. Concentrations of toxicants were transformed in logarithms, and four different models (power series, polynomials, exponential models and Fourier series) were then used to construct the dose–response curves and calculate the IC_5_ and IC_50_ values [44,45]. A nonlinear least squares matching procedure was used to select the most fitted equation and to reach a more accurate correspondence of fit to the data points [46].

**Figure 1:**
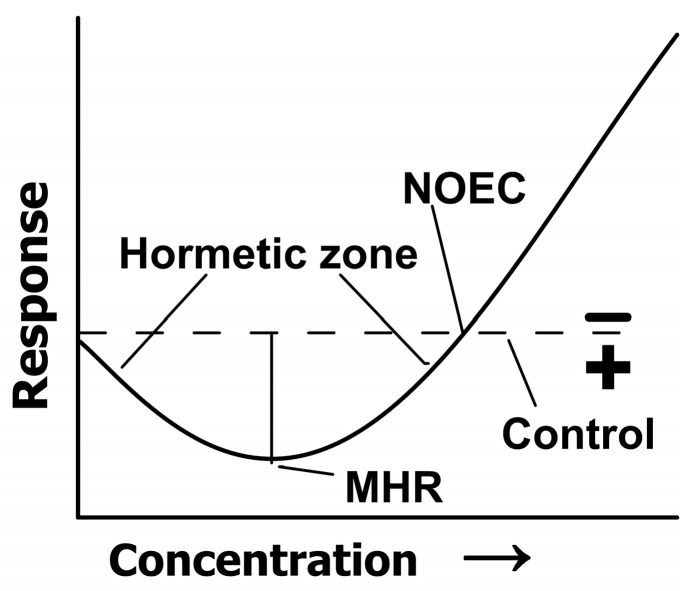
Dose–response relationship depicting the quantitative feature of hormesis [33]. The signs + and − indicate biologically positive and negative effects, respectively.

The important indexes of hormetic-like response are the no-observed-adverse-effect-level (NOAEL) or concentration (NOEC, i.e., the dose where the response crosses the control level or zero equivalent point) and the maximum hormetic response (MHR, %) [33,47] (Figure 1). The values of IC_5_, IC_50_, NOEC and MHR were calculated according to regression equations.

Nonparametric Kruskall–Wallis test was used to determine statistical significance of the differences between toxic effects of investigated lanthanides [44]. The differences were considered significant when *p* < 0.05.

The statistical analysis was performed with the MATLAB (R2022a) statistical software package.

## 3. Results

### 3.1. Tolerance of Microscopic Fungi to Different Concentrations of La, Ce and Nd

In total, 15 species of microscopic fungi were identified from the tailing dump of the Lovozersky MPP [26,38]. The dominant species were *G. vinaceus* (20% of relative abundance), *P. simplicissimum* (18%) and *U. isabellina* (14%). The relative abundance of other species, including *A. niveoglaucus* (9%) and *P. pannorum* (7%), did not exceed ten percent. Five isolated species (*G. vinaceus*, *P. simplicissimum*, *U. isabellina*, *A. niveoglaucus* and *P. pannorum*) and the control one, *S. polyspora*, were chosen to test their tolerance to lanthanides (La, Ce and Nd). The degree of REE tolerance exhibited by fungi was determined based on the growth of strains in the presence of lanthanides in the form of chloride and nitrate salts. The colony diameters were measured daily at different REEs’ concentrations (0~600 mg/L). Different strains exhibited different responses to lanthanides in the nitrate and chloride forms (Table S1). For instance, the inhibition ratio of *G. vinaceus* was only 16% when the concentration of Ce^3+^ in the form of nitrate was 216 mg/L, and its growth completely stopped when the concentration of metal was 142 mg/L in the case of chloride (Table S1). A similar growth behavior was observed for *P. pannorum*. All isolates and *S. polyspora* showed resistance to different lanthanides over a wide range of REE concentrations (5.5–200 mg/L) in the presence of nitrates. In the case of nitrates, almost all fungi except *P. simplicissimum* stopped their growth when the concentration of lanthanides was over 424 mg/L (La^3+^), 432 mg/L (Ce^3+^) and 440 mg/L (Nd^3+^) (Table S1). However, the presence of chlorides lowered the threshold of microfungal sensitivity to lanthanides.

**Figure 2:**
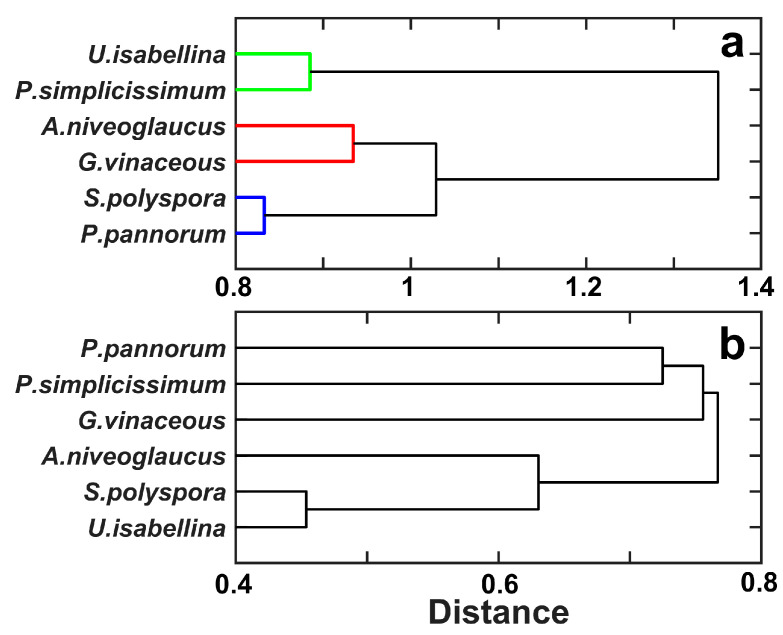
Dendrogram based on the Euclidean distance and the nearest-neighbor clustering method showing the different growth patterns of the selected microfungi exposed to REEs in the form of chlorides (**a**) and nitrates (**b**). Each color group in the dendrogram combines similar elements whose linkage is less than the threshold value (70% of the maximum linkage).

The dendrogram of fungal clustering obtained by the nearest-neighbor linkage method, which took into account the results of fungal growth responses to Ce^3+^ and Nd^3+^, demonstrated two major clusters in the case of chloride solution (Figure 2a). The first cluster included the dominant isolates *P. simplicissimum* and *U. isabellina*, thereby confirming its considerable difference from all other strains. The second major cluster was subdivided into two subgroups. The first subgroup combined the isolates *A. niveoglaucus* and *G. vinaceus*, while the second subgroup contained the non-dominant isolate *P. pannorum* and the control strain *S. polyspora* (Figure 2a). In the presence of chlorides, these two strains were less resistant to lanthanides compared to other fungi. However, in the presence of nitrates, the benefit of *P. simplicissimum* and *U. isabellina* in lanthanide tolerance was lost, and the difference between other species disappeared (Figure 2b).

### 3.2. Hormesis and Low-Dose Toxicity of REEs

Low toxic effects of lanthanides (La^3+^, Ce^3+^, Nd^3+^) were assessed. In order to determine the IC_5_ and IC_50_ values, we used the following equations:

(2) $$y=a_{0}+a_{1}x^{b}\;[\mathrm{power}],$$

(3) $$y=a_{0}+\sum_{k=1}^{n}a_{k}x^{k}\;[\mathrm{polynomial}],$$

(4) $$y=a_{0}+\sum_{k=1}^{n}a_{k}e^{b_{k}x}\;[\mathrm{exponential}],$$

(5) $$y=a_{0}+\sum_{k=1}^{n}\left[a_{k}\cos\left(kwx\right)+b_{k}\sin\left(kwx\right)\right]\;[\mathrm{Fourier}],$$

(6) y=a0+c−bxa [Hill equation],

(7) $$y=a_{0}+\sum_{k=1}^{n}a_{k}x^{k}+\sum_{k=1}^{n}b_{k}e^{c_{k}x}+\sum_{k=1}^{n}d_{k}\sin\left(kwx\right)+fx^{m}\;[\mathrm{mixt}],$$

where $a_{0}$, $a_{k}$, $b_{k}$, $c_{k}$, $d_{k}$, *a*, *b*, *f*, *m* and *w* are parameters to be determined by fitting with the data points, and *x* is the exposure concentration (in log units).

**Figure 3:**
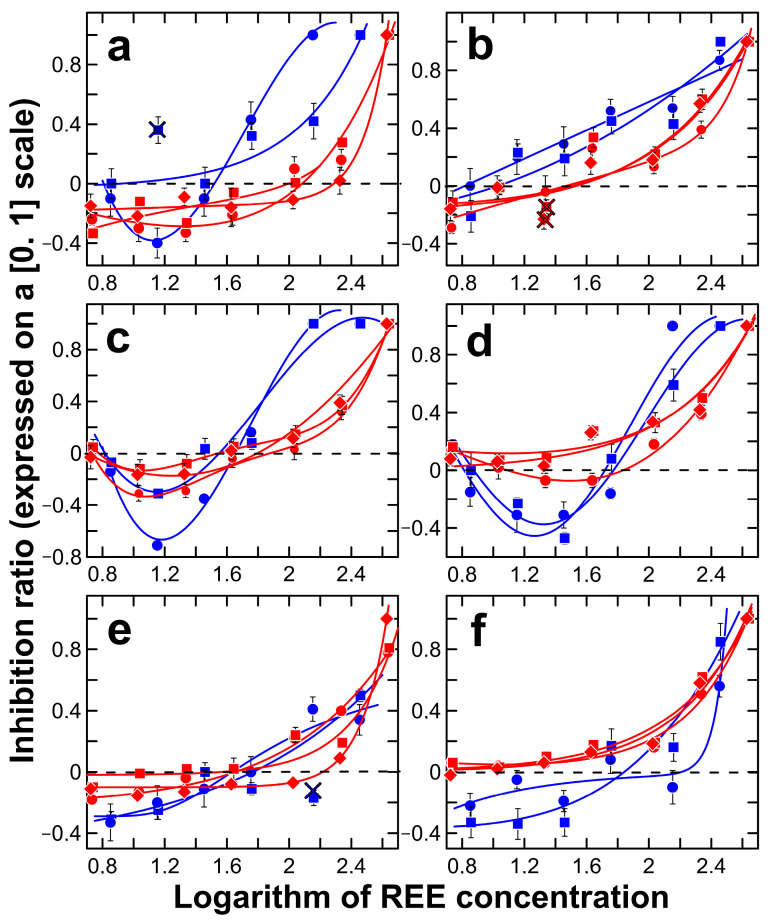
Concentration–response curves of Nd (squares), Ce (circles) and La (for nitrates only, diamonds) toxicity as chlorides (blue) and nitrates (red) to selected fungi: *G. vinaceous*—(**a**), *A. niveoglaucus*—(**b**), *P. pannorum*—(**c**), *S. polyspora*—(**d**), *P. simplicissimum*—(**e**) and *U. isabellina*—(**f**). Growth inhibition ratio IR (%) is expressed on a [0, 1] scale. Data are averages of three replicates with error bars showing the standard error of the mean. Outliers are depicted by crosses. Dashed line denotes the control level.

The concentration–response data and regression curves obtained for chlorides (blue) and nitrates (red) of Nd, Ce and La (for nitrates only) on the basis of different models are reported in Figure 3. Figure 3 shows the results of the inhibitory effect of lanthanides on selected fungi, including *A*. *niveoglaucus*, *G*. *vinaceus*, *P*. *simplicissimum*, *P. pannorum* and *Sydowia polyspora*, after an incubation period of 12 days and *U. isabellina* after an incubation period of seven days in the dark at 20 °C. Table 1 shows the regression parameters, coefficient of determination *R*^2^, the IC_5_ and IC_50_ values (mg/L) and their relative limits of confidence at 95% probability (mg/L). NOEC (mg/L) and MHR (%) are given for the case of the hormetic effect (Table 1). *R*^2^ values confirmed the goodness of fit with selected regressions, showing values ranging from 0.8488 to 0.9996 (Table 1).

As one can see, the fungal growth was inhibited (positive IR values) by high levels of lanthanides, but it was stimulated (negative IR values), with some exceptions, at low treatment levels, showing a hormetic effect (Figure 3 and Table 1). All metals caused the hormetic response to one degree or another on each strain. Our findings indicate that the maximum hormetic response (MHR) was greater than 12% and less than 67% as compared to the control value (Table 1). For instance, negative IR values indicated the stimulation of *P. pannorum* growth at low concentrations (NOEC = 46 mg/L) with the highest MHR value of 67% and inhibition at high levels of Ce^3+^ in the form of chloride (Figure 3c and Table 1). In a few cases, *P. simplicissimum* (La(NO_3_)_3_, Nd(NO_3_)_3_), *A. niveoglaucus* (CeCl_3_, NdCl_3_) and *S. polyspora* (Ce(NO_3_)_3_) demonstrated a slight (MHR < 10%) hormesis-like response that was statistically insignificant [32,33] (Figure 3b–e and Table 1). All our other cases satisfied the criteria for evaluating hormesis with a high degree of evidence (acceptable dose range (>100-fold) and number of doses (six–seven) with three–four doses in the hypothetical hormetic zone (i.e., doses less than the NOEC)) [32]. And only two microfungal species, *S. polyspora* and *U. isabelline*, demonstrated the absence of a hormetic-like response when exposed to metals as nitrates at low doses, while these strains showed a statistically significant hormesis at low concentrations in the case of chlorides (Figure 3d,f and Table 1).

**Figure 4:**
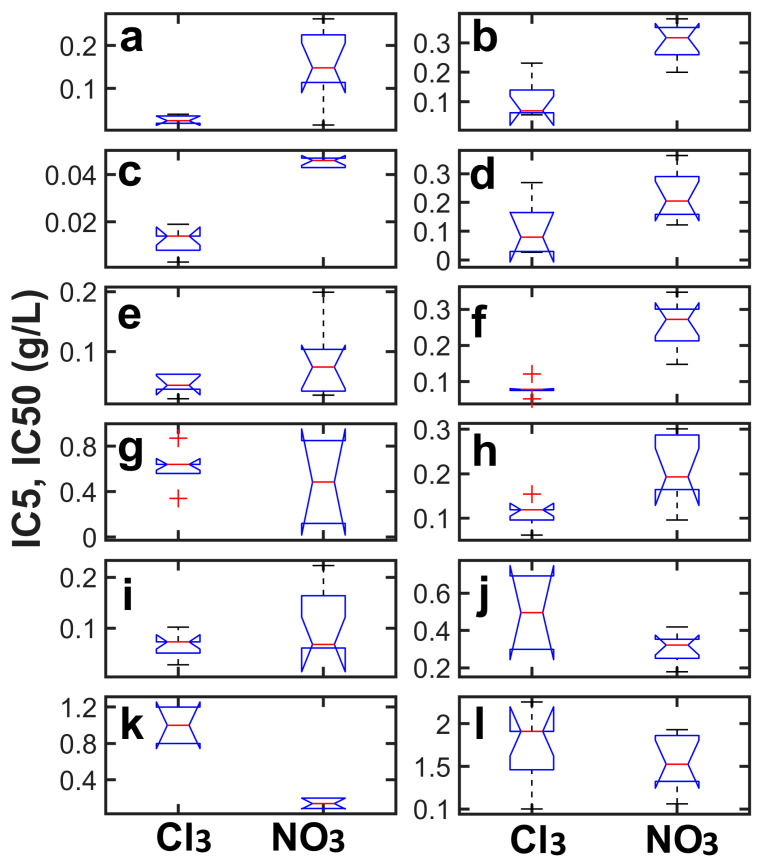
Results of nonparametric Kruskall–Wallis test applied to determine statistical significance of the differences between toxic effects at low (IC_5_; the left part of the panel) and medium (IC_50_, the right part of the panel) levels of investigated lanthanides in chloride and nitrate form to selected fungi: *G. vinaceous*—(**a**,**b**), *A. niveoglaucus*—(**c**,**d**), *P. pannorum*—(**e**,**f**), *S. polyspora*—(**g**,**h**), *P. simplicissimum*—(**i**,**j**) and *U. isabellina*—(**k**,**l**). On each box, the central red line indicates the median. Outliers are marked with red pluses.

Differences between chlorides and nitrates at low (IC_5_) toxic levels were statistically significant (*p* < 0.05) only for *G. vinaceous* (Figure 4a), *A. niveoglaucus* (Figure 4c) and *U. isabellina* (Figure 4k). Interestingly, in the presence of nitrates, lanthanides showed a significantly (*p* = 0.0012) higher inhibition ratio and low-dose toxicity (IC_5_ = 9–20 mg/L) compared to chlorides (IC_5_ = 80–195 mg/L) for *U. isabellina* (Figure 4k and Table 1). Lanthanides demonstrated a similar toxicity at low doses, showing IC_5_ values ranging from 8 to 195 mg/L for Ce^3+^, from 14 to 150 mg/L for Nd^3+^ and from 12 to 218 mg/L for La^3+^ (Table 1). In addition, the differences between the low-dose toxicity values of individual REEs were statistically significant (*p* < 0.05) only for some species. *G. vinaceous* and *U. isabellina* showed a higher (*p* < 0.05) toxicity of Nd^3+^ compared to Ce^3+^ in the chloride form. Interestingly, *U. isabellina* and the other two species (*P. simplicissimum* and *A. niveoglaucus*) demonstrated an opposite response at low doses of REEs in the case of nitrates. So, the toxicity of lanthanides in nitrate form at low doses in decreasing order (*p* < 0.05) was as follows: Ce^3+^ > Nd^3+^ > La^3+^.

The medium toxic effects of metal ions in the chloride form (IC_50_ about 63–96 and 78–140 mg/L in the case of Ce^3+^ and Nd^3+^, respectively) were significantly (*p* < 0.05) higher compared to nitrates (IC_50_ about 234–286 and 193–300 mg/L in the case of Ce^3+^ and Nd^3+^, respectively) for four fungal species (*G. vinaceous*, *A. niveoglaucus*, *P. pannorum* and *S. polyspora*; Table 1 and Figure 4b,d,f,h). The difference between medium toxic effects of REEs such as nitrates and chlorides was statistically insignificant only for *P. simplicissimum* and *U. isabellina* (Figure 4j and Figure 4l, respectively). The difference between the medium-dose toxic effects of individual lanthanides was significant (*p* < 0.05) only for *P. simplicissimum*, showing a higher toxicity of Nd^3+^ compared to Ce^3+^ in the chloride form.

## 4. Discussion

In the present study, the low-dose toxic effects of chloride and nitrate salts of three REEs (Ce^3+^, Nd^3+^ and La^3+^) were tested on six microfungal species. Five of them (*G. vinaceous*, *A. niveoglaucus*, *P. pannorum*, *P. simplicissimum* and *U. isabellina*) were isolated from the Lovozersky MPP’s loparite ore concentration tailings at the Kola Peninsula. *S. polyspora* was chosen as a control strain. All these species are widely spread in the natural and polluted soils of the Kola Peninsula [26,48,49]. The choice of La, Ce and Nd for study purposes was due to the relatively significant content of these REEs in the wastes of the loparite and apatite–nepheline mining industry, which are the main sources of negative environmental impact on the Kola Peninsula [11,12,13]. Additionally, selected lanthanides and nitrates are widely used in REE-enriched fertilizers [27]. In our previous reports, we have tested these species for Ce and Nd tolerance [19,26]. As a result, the most tolerant to both metals were *P. simplicissimum* and *U. isabellina*, which continued to grow at the highest (500 mg/L) concentrations of CeCl_3_ and NdCl_3_ [19,26]. Several researchers reported similar microfungal growth behavior in the presence of Nd^3+^ [25] and La [50,51]. For instance, the growth of the pathogenic fungus *Rhizoctonia solani* was completely inhibited when the concentration of La in the media was 600 mg/L [50]. The growth of *Fusarium oxysporum* was inhibited when Nd^3+^ concentration was greater than 200 mg/L [25]. According to our present findings, in the presence of chlorides, the dominant isolates, *P. simplicissimum* and *U. isabellina*, were resistant to much higher concentrations of REEs compared to other strains. However, in the presence of nitrates, such a feature in the growth behavior of these dominant isolates disappeared. Tolerance studies can help us to test the ability of microfungal isolates to survive at high REE concentrations and, therefore, assess their possible bioremediation (or bioleaching) potential. The most important mechanisms of fungal tolerance to REEs (or potential bioleaching) are the following: chemical transformation or dissolving of components by the organic acids secreted by fungi; bioaccumulation or energy-dependent flux into the cell; and biosorption or metabolism-independent binding to the cell surface [52,53]. To date, *P. simplicissimum* fungus is one of the most common species used for metal bioleaching applications [52,53,54]. However, only a few studies tested the tolerance of *U. isabellina* to heavy metals [55] and REEs [26].

The aim of the present study was to assess the low-dose toxic effect of three lanthanides (La, Ce and Nd) and to test whether REE chlorides and nitrates were different in this context. According to our findings, the REE toxicity in the presence of chlorides was significantly (*p* < 0.05) higher compared to nitrates for *G. vinaceous* and *A. niveoglaucus* (Figure 4a–d). A similar relationship was found for *P. pannorum* and *S. polyspora* only at a medium level of toxicity (Figure 4f,h). Interestingly, *U. isabellina* only showed an opposite response, indicating the highest toxicity of nitrate (IC_5_ = 9–20 mg/L) REEs’ salts compared to chlorides (IC_5_ = 80–195 mg/L) at low concentration levels (Figure 4k and Table 1). A similar but nonsignificant behavior of *U. isabellina* fungus was observed at the medium level of toxicity (Figure 4i).

Lanthanides are a chemically uniform group of metals; nevertheless, the light lanthanides (La–Eu) have an ionic radius close to that of Ca^2+^ [20,56]. Therefore, the mass replacement of Ca^2+^ by lanthanides can block calcium ionic channels in microorganisms and may disturb the uptake of nutrients through these channels. The lower toxicity of REEs in the presence of nitrate salts could be explained by a “joint effect” of the metal (Ce^3+^, Nd^3+^ and La^3+^) and nitrate ions, which can affect fungal growth in the opposite way, as was shown earlier for other elements [57,58]. In this case, it is possible to hypothesize that nitrates can play a role in nutrition as a nitrogen source, compensating for the toxic effect of REEs at low and medium levels. Therefore, in the case of nitrate salts toxic effects of lanthanides were observed at higher concentrations compared to chlorides. It is known that lanthanides may form hardly soluble complexes and precipitate with organic or inorganic ligands, thus decreasing their availability and toxicity potential [24,36,56,59]. Indeed, the presence of anions such as phosphate or sulfate in the culture medium was recently reported to decrease the REE bioavailability and toxicity [24,59]. However, there is no difference in the solubility of chloride or nitrate REE salts [56]. In the present study, the observed differences in toxicity between lanthanides in the nitrate and chloride forms would not, therefore, be related to differences in the solubility and subsequent REE availability in different culture media.

All three lanthanides showed concentration-related hormetic trends with growth stimulation at low doses and inhibition at high doses. The highest (MHR = 67%) hormetic response with NOEC = 46 mg/L occurred for *P. pannorum* when exposed to Ce^3+^ in the presence of chlorides (Table 1). The hormetic effect was also observed in other species with a lower power. Our findings indicate that REEs in the chloride form exhibited a greater hormetic effect compared to the nitrate salts. In other words, the presence of nitrate ions suppressed the development of hormesis in selected fungal species after exposure to REEs. Contrarily, *U. isabellina* and *S. polyspora* demonstrated a different behavior compared to other fungi, i.e., the absence of hormetic dose responses after the exposure to lanthanides in the presence of nitrates (Figure 4d,f and Table 1). This may be due to different metabolic processes and/or to different adaptive capabilities among fungal species under the REE treatment.

*S. polyspora* is reported as a plant pathogen infecting conifers around the world [60]. A unique feature of *U. isabellina* is its superior capability for microbial lipids accumulation over other oleaginous microorganisms [61]. These microbial lipids are the potential feedstock for lipid-based biofuel production [61]. According to our previous findings, only *U. isabellina*, among other isolates, started to grow after extreme exposure to 1000 mg/L of CeCl_3_ one month after inoculation [26]. In fact, the growth of *U. isabellina* at the highest concentrations of 500 mg/L and 1000 mg/L CeCl_3_ was accompanied by the production of lipid-rich chlamydospores (or thick-walled asexual spores), which are well adapted to maintaining fungus viability under stress conditions [26,62]. Our present findings indicate that the absorption of additional nitrogen can negate the adaptive processes in *U. isabellina* at low doses of lanthanides. Indeed, the high nitrogen content sets a low carbon-to-nitrogen (C/N) ratio in *U. isabellina*, which can have negative effects on lipid production in the strain [62].

Despite the absence of a single mechanism explaining the hormesis phenomenon, there is notable evidence indicating its adaptive nature with an ability to regulate antioxidative enzymatic processes [33,34]. On the other hand, hormesis may also be associated with the increased uptake of nutrients, including nitrogen [63]. In the case of *U. isabellina* and *S. polyspora*, the uptake of additional nitrogen can negate adaptive processes in fungi in the hormetic zone of concentrations. However, the physiological mechanisms of hormetic response are still not well understood [33,34].

## 5. Conclusions

To the best of our knowledge, this is the first report on the hormesis and low-dose toxic effects of lanthanides (La, Ce and Nd) on the fungi which were isolated from Arctic loparite ore concentration tailings. For all the tested REEs, the highest concentrations led to noticeable growth inhibition, indicating that all lanthanides were toxic to fungal isolates. The results showed that in the presence of nitrates, the toxicity of Ce^3+^ and Nd^3+^ to four of six fungal isolates was significantly (*p* < 0.5) lower compared to chlorides. In this case, nitrates can play the role of exogenous nutrients, compensating for the toxic effect of REEs.

In addition, the toxicity of La, Ce and Nd had a hormetic effect on fungal growth with stimulation at low doses and inhibition at high doses. Contrarily, *U. isabellina* and *S. polyspora* demonstrated the absence of hormetic response under treatment with REEs’ nitrate salt. Two distinct hormesis dose–response patterns first identified in fungal isolates may contribute to a better understanding of the hormetic and toxicity processes associated with lanthanide exposure. Taking into account a high tolerance of *P. simplicissimum* and *U. isabellina* and a high pathogenicity of *S. polyspora* for coniferous trees, our findings on the hormetic responses may be useful in the assessment of the potential application of the isolated fungi to bioremediation and REE bioleaching, and, as in the case of *S. polyspora*, to the reforestation of REE-contaminated areas.

## Figures and Tables

**Table 1:** Effects of REEs at hormetic and toxic dose on fungi.

Species	REEs	Regression Equation	*R* ^2^	IC_5_ (mg/L)	IC_50_ (mg/L)	Hormetic/ Toxic Effect *
*G. vinaceus* nitrate	La^3+^	Equation (7): *n* = 1, *a*_0_ = −0.2, *a*_1_ = 0, *b*_1_ = 5.56 × 10^−8^, *c*_1_ = 6.4, *d*_1_ = −1, *w* = −0.03222, *f* = 0	0.9923	218 (148, 262)	343 (317, 366)	Hormetic effect: NOEC = 194; MHR > 17
	Ce^3+^	Equation (7): *n* = 1, *a*_0_ = 0, *a*_1_ = 0, *b*_1_ = 5.5684 × 10^−7^, *c*_1_ = 1.657, *d*_1_ = −1, *w* = −0.39, *f* = 0	0.9613	135 (81, 195)	275 (213, 347)	Hormetic effect: NOEC = 121; MHR = 29
	Nd^3+^	Equation (4): *n* = 2, *a*_0_ = 0, *a*_1_ = 4.48 × 10^−5^, *a*_2_ = −0.6271, *b*_1_ = 3.805, *b*_2_ = −0.9829	0.9801	125 (15, 245)	300 (200, 381)	Hormetic effect: NOEC = 100; MHR = 33
chloride	Ce^3+^	Equation (3): *n* = 3, *a*_0_ = 6.257, *a*_1_ = −14.09, *a*_2_ = 9.318, *a*_3_ = −1.813	0.9996	36 (31, 40)	63 (56, 71)	Hormetic effect: NOEC = 33; MHR = 40
	Nd^3+^	Equation (4): *n* = 1, *a*_0_ = −0.03, *a*_1_ = 3.796 × 10^−3^, *b*_1_ = 2.269	0.9556	19	140 (68, 231)	Toxic effect
*A. niveoglaucus* nitrate	La^3+^	Equation (4): *n* = 1, *a*_0_ = −0.19, *a*_1_ = 0.01441, *b*_1_ = 1.683	0.945	47	199 (122, 296)	Hormetic effect: NOEC = 34; MHR = 14
	Ce^3+^	Equation (7): *n* = 1, *a*_0_ = −0.46, *a*_1_ = 0, *b*_1_ = 5.684 × 10^−7^, *c*_1_ = 5.323, *d*_1_ = −1, *w* = −0.3255, *f* = 0	0.9835	43	277 (168, 363)	Hormetic effect: NOEC = 29; MHR > 25
	Nd^3+^	Equation (4): *n* = 1, *a*_0_ = −0.164, *a*_1_ = 0.01167, *b*_1_ = 1.748	0.9781	46	205 (131 288)	Hormetic effect: NOEC = 32; MHR = 13
chloride	Ce^3+^	Equation (3): *n* = 1, *a*_0_ = −0.4016, *a*_1_ = 0.492	0.9499	8 (3, 19)	68 (30, 165)	Hormetic effect (MHR < 10%) **
	Nd^3+^	Equation (3): *n* = 2, *a*_0_ = −0.19, *a*_1_ = 0, *a*_2_ = 0.1797	0.8488	14	91 (27, 269)	Hormetic effect (MHR < 10%) **
*P. simplicissimum* nitrate	La^3+^	Equation (4): *n* = 1, *a*_0_ = −0.1, *a*_1_ = 2.372 × 10^−7^, *b*_1_ = 5.843	0.9958	193 (154, 223)	333 (316, 350)	Hormetic effect (MHR < 10%) **
	Ce^3+^	Equation (4): *n* = 2, *a*_0_ = 0, *a*_1_ = −0.221, *a*_2_ = −0.02294, *b*_1_ = 0.0824, *b*_2_ = 1.452	0.9867	61	263 (179, 362)	Hormetic effect: NOEC = 45; MHR > 17
	Nd^3+^	Equation (4): *n* = 1, *a*_0_ = −0.02, *a*_1_ = 1.009 × 10^−4^, *b*_1_ = 3.392	0.9315	68	322 (215, 419)	Hormetic effect (MHR < 10%) **
chloride	Ce^3+^	Equation (6): *a*_0_ = −0.29, *a* = 3, *b* = 3, *c* = 6.05	0.9335	51 (28, 102)	692	Hormetic effect: NOEC = 33; MHR = 40
	Nd^3+^	Equation (7): *n* = 1, *a*_0_ = 0, *a*_1_ = 0, *b*_1_ = 0.08238, *c*_1_ = 1, *d*_1_ = 0, *f* = −0.49, *m* = −0.02888	0.9294	73	299	Hormetic effect: NOEC = 59; MHR > 31
*P. pannorum* nitrate	La^3+^	Equation (3): *n* = 2, *a*_0_ = 0.72, *a*_1_ = −1.434, *a*_2_ = 0.5752	0.967	74	214 (148, 299)	Hormetic effect: NOEC = 62; MHR = 17
	Ce^3+^	Equation (3): *n* = 4, *a*_0_ = 6.224, *a*_1_ = −17.59, *a*_2_ = 16.92, *a*_3_ = −6.915, *a*_4_ = 1.04	0.9948	98 (33, 199)	286 (209, 347)	Hormetic effect: NOEC = 72; MHR = 33
	Nd^3+^	Equation (3): *n* = 4, *a*_0_ = 4.031, *a*_1_ = −11.45, *a*_2_ = 11.31, *a*_3_ = −4.759, *a*_4_ = 0.7393	0.9994	49 (27, 89)	272 (237, 304)	Hormetic effect: NOEC = 33; MHR = 13
chloride	Ce^3+^	Equation (3): *n* = 3, *a*_0_ = 8.882, *a*_1_ = −19.5, *a*_2_ = 12.47, *a*_3_ = −2.375	0.9961	48 (37, 62)	76 (53, 121)	Hormetic effect: NOEC = 46; MHR = 67
	Nd^3+^	Equation (3): *n* = 3, *a*_0_ = 4.5, *a*_1_ = −9.882, *a*_2_ = 6.3, *a*_3_ = −1.16	0.9428	39 (21, 62)	78 (50, 130)	Hormetic effect: NOEC = 36; MHR = 29
*S. polyspora* nitrate	La^3+^	Equation (4): *n* = 1, *a*_0_ = 0, *a*_1_ = 6.79 × 10^−3^, *b*_1_ = 1.883	0.9466	12	192 (103, 288)	Toxic effect
	Ce^3+^	Equation (3): *n* = 3, *a*_0_ = 0.3549, *a*_1_ = −0.50327, *a*_2_ = −0.5037, *a*_3_ = 0.2329	0.9912	85	234 (185, 287)	Hormetic effect (MHR < 10%) **
	Nd^3+^	Equation (4): *n* = 2, *a*_0_ = 0, *a*_1_ = 0.2992, *a*_2_ = 5.709 × 10^−3^, *b*_1_ = −1.338, *b*_2_ = 1.943	0.9725	_	193 (96, 301)	Toxic effect
chloride	Ce^3+^	Equation (5): *n* = 1, *a*_0_ = 0.31, *a*_1_ = 0.762, *b*_1_ = 0.06441, *w* = 2.55	0.9412	56 (34, 87)	96 (62, 154)	Hormetic effect: NOEC = 52; MHR = 46
	Nd^3+^	Equation (5): *n* = 1, *a*_0_ = 0.3363, *a*_1_ = 0.7105, *b*_1_ = 0.02512, *w* = 2.4	0.9741	64	119	Hormetic effect: NOEC = 59; MHR = 37
*U. isabellina* nitrate	La^3+^	Equation (4): *n* = 1, *a*_0_ = 0, *a*_1_ = 2.69 × 10^−3^, *b*_1_ = 2.259	0.9857	20	205 (143, 268)	Toxic effect
	Ce^3+^	Equation (4): *n* = 2, *a*_0_ = 0, *a*_1_ = 0.1587, *a*_2_ = 1.183 × 10^−3^, *b*_1_ = −1.557, *b*_2_ = 2.559	0.9916	9	229 (172, 285)	Toxic effect
	Nd^3+^	Equation (4): *n* = 1, *a*_0_ = 0, *a*_1_ = 5.004 × 10^−3^, *b*_1_ = 2.008	0.9733	14	196 (112, 286)	Toxic effect
chloride	Ce^3+^	Equation (4): *n* = 2, *a*_0_ = 0, *a*_1_ = 2.558 × 10^−15^, *a*_2_ = −0.83, *b*_1_ = −13.47, *b*_2_ = −1.66	0.8988	195	279 (247, 309)	Hormetic effect: NOEC = 158; MHR > 23
	Nd^3+^	Equation (2): *a*_0_ = −0.367, *a*_1_ = 0.03188, *b* = 4.	0.9132	80	192 (100, 351)	Hormetic effect: NOEC = 69; MHR > 35

* Values of NOEC and MHR are depicted in mg/L and (%), respectively. ** Slight or insignificant (MHR < 10%) effect that does not satisfy the criteria for evaluating hormesis [32,33].

## Data Availability

Data are contained within the article and supplementary materials.

## Supplementary Materials

The following supporting information can be downloaded at: https://www.mdpi.com/article/10.3390/toxics11121010/s1, Table S1: Inhibition ratio (IR) shown by microfungal strains at different concentrations of REEs in the presence of nitrates and chlorides.
