# Supplementary material for: Hormesis and Low Toxic Effects of Three Lanthanides in Microfungi Isolated from Rare Earth Mining Waste in Northwestern Russia
Source: Toxics. 2023 Dec 10;11(12):1010. doi: 10.3390/toxics11121010 (PMC10747132; doi:10.3390/toxics11121010)
Supplement: Supplementary file 1 [file toxics-11-01010-s001.zip › toxics-2732083-supplementary.pdf]

Table S1. Inhibition ratio (IR) shown by microfungal strains at different concentrations of REEs in the presence of nitrates and chlorides.

Species	Bank Accession Number	Relative abundance (%)	REEs	Concentration (mg/L)		IR (%)	
				Nitrate	Chloride	Nitrate	Chloride
Geomyces vinaceus	OQ165231	20	Nd^{3+}	5.5	7	-34	0
				11	14	-12	36
				22	29	-27	0
				44	58	-6	32
				110	144	1	42
				220	288	28	100
				440	576	100	100
			Ce^{3+}	5.5	7	-24	-10
				11	14	-30	-40
				22	28	-33	-10
				43	57	-21	43
				108	142	10	100
				216	284	16	100
				432	568	100	100
			La^{3+}	5.5		-15	
				11		-22	
				21		-9	
				42		-16	
				106		-11	
				212		2	
				424		100	
Aspergillus niveoglaucus	OQ165229	9	Nd^{3+}	5.5	7	-11	-21
				11	14	-1	23
				22	29	-15	19
				44	58	34	45
				110	144	22	43
				220	288	60	100
				440	576	100	100
			Ce^{3+}	5.5	7	-29	0
				11	14	-3	20
				22	28	-4	29
				43	57	26	52
				108	142	14	54
				216	284	39	87
				432	568	100	100
			La^{3+}	5.5		-16	
				11		-1	
				21		-23	
				42		16	
				106		18	
				212		57	
				424		100	
			Nd^{3+}	5.5	7	-10	-31
				11	14	-1	-25
				22	29	2	0
				44	58	2	-11

				110	144	24	-17
				220	288	19	50
				440	576	81	100
			Ce^{3+}	5.5	7	-18	-33
				11	14	-13	-20
				22	28	-4	-11
Penicillium simplicissimum	OQ165232	18		43	57	-8	0
				108	142	22	41
				216	284	40	34
				432	568	78	100
			La^{3+}	5.5		-11	
				11		-16	
				21		-13	
				42		-8	
				106		-7	
				21		9	
				424		80	
			Nd^{3+}	5.5	7	6	-33
				11	14	3	-34
				22	29	10	-33
				44	58	18	17
				110	144	19	16
				220	288	62	85
				440	576	100	100
			Ce^{3+}	5.5	7	6	-22
				11	14	4	-5
				22	28	6	-19
Umbelopsis isabellina	OQ165236	14		43	57	12	8
				108	142	16	-10
				216	284	51	56
				432	568	100	100
			La^{3+}	5.5		-2	
				11		2	
				21		6	
				42		13	
				106		19	
				212		58	
				424		100	
			Nd^{3+}	5.5	7	5	-7
				11	14	-12	-31
				22	29	-8	3
				44	58	5	8
				110	144	15	100
				220	288	37	100
				440	576	100	100
			Ce^{3+}	5.5	7	0	-14
				11	14	-31	-71
Pseudogymnoascus pannorum		7		22	28	-29	-35
				43	57	-4	16
				108	142	3	100
				216	284	32	100
				432	568	100	100
			La^{3+}	5.5		-3	

			11		-17	
			21		-16	
			42		2	
			106		12	
			212		39	
			424		100	
		Nd^{3+}	5.5	7	16	0
			11	14	7	-23
			22	29	9	-47
			44	58	27	8
			110	144	33	59
			220	288	50	100
			440	576	100	100
		Ce^{3+}	5.5	7	14	-15
			11	14	2	-31
			22	28	-7	-31
Sydowia polyspora	Control		43	57	-7	-16
			108	142	18	100
			216	284	39	100
			432	568	100	100
		La^{3+}	5.5		8	
			11		6	
			21		3	
			42		26	
			106		34	
			212		42	
			424		100	
